# Probability, Entropy, and Gibbs’ Paradox(es)

**DOI:** 10.3390/e20060450

**Published:** 2018-06-09

**Authors:** Robert H. Swendsen

**Affiliations:** Physics Department, Carnegie Mellon University, Pittsburgh, PA 15213, USA; swendsen@cmu.edu; Tel.: +1-412-268-5211

**Keywords:** Gibbs’ paradox, statistical mechanics, entropy

## Abstract

Two distinct puzzles, which are both known as Gibbs’ paradox, have interested physicists since they were first identified in the 1870s. They each have significance for the foundations of statistical mechanics and have led to lively discussions with a wide variety of suggested resolutions. Most proposed resolutions had involved quantum mechanics, although the original puzzles were entirely classical and were posed before quantum mechanics was invented. In this paper, I show that contrary to what has often been suggested, quantum mechanics is not essential for resolving the paradoxes. I present a resolution of the paradoxes that does not depend on quantum mechanics and includes the case of colloidal solutions, for which quantum mechanics is not relevant.

## 1. Introduction

Among the conceptual difficulties encountered by early workers on the statistical foundations of thermodynamics, the problems that have come to be known as Gibbs’ paradox (or paradoxes) are among the most famous. In this paper, I give an interpretation of the origin of the paradoxes and their resolution. Although quantum mechanics has often been suggested as essential to the resolution of these paradoxes, I do not believe that quantum mechanics is necessary, or even relevant, to the discussion.

The first paradox was noted by Gibbs in 1875 [1]. It concerned different expressions for the entropy change from the mixing of two ideal gases, depending on whether the gases were the same or different. If the two gases were the same, Gibbs found no change in total entropy. If the two gases were different, even if the difference was very small, there was a change in the total entropy that depended only on the number of particles in each gas, but not on the nature of the gases themselves. It was especially disturbing that the difference in entropy vanished discontinuously as the difference in the two gases went to zero. The lack of complete understanding of the mixing of two gases was regarded as an indication that there might be something fundamentally wrong with the idea of entropy [2,3]. I will denote this puzzle as the first Gibbs’ paradox.

The second puzzle, which is also known as Gibbs’ paradox, arose in connection with a misinterpretation of Boltzmann’s 1877 definition of the entropy [4,5]. It was assumed that Boltzmann had defined entropy as being proportional to an accessible volume in phase space of an isolated thermodynamic system. This definition led to an expression for the entropy of an ideal gas that was not extensive because it lacked a term proportional to ln(1/N!), where *N* is the number of particles in the system. As Gibbs had pointed out, if this term is omitted from the formula, the entropy of a mixture of two gases of the same kind gives a spurious extra term [6]. The origin of the factor of 1/N! is still subject to debate. I will denote it as the second Gibbs’ paradox.

The factor 1/N! in the definition of the entropy is most commonly attributed to the quantum nature of matter [7]. I hope to show in this paper that neither the first nor the second Gibbs’ paradox has anything to do with quantum mechanics; their resolutions require only classical theory. I will make no use of quantum concepts in my arguments.

The resolution of Gibbs’ paradoxes requires an explicit statement of the assumptions forming the basis of the theory of statistical mechanics. Unfortunately, such assumptions are not universally accepted, but I will try to express mine clearly to serve as a basis for discussion. On the other hand, the derivation of most equations in statistical mechanics does not require such a precise exposition—at least if the arguments are not examined too closely. Because even incorrect arguments can lead to (mostly) correct results, some dubious assumptions have become generally accepted and must be challenged in order to achieve a final resolution of Gibbs’ paradoxes.

The entropy is a central concept in thermodynamics, which makes it necessary to specify thermodynamics explicitly, which is done in the next section.

## 2. What is Thermodynamics?

First, the domain of thermodynamics must be specified, then the role of limited resolution, and the purpose of the theory. The postulates of thermodynamics provide a convenient list of properties that the entropy must satisfy [8,9,10,11,12].

### 2.1. The Domain of Thermodynamics

Thermodynamics is defined on the set of all finite, macroscopic systems that might exchange energy, volume, or particles with each other. The number of such systems, *M*, is very large, but finite. The prevention of any two systems from exchanging energy, volume, or particles is called a constraint. Constraints can be either imposed or released, as the experimenter wishes.

Denoting the *j*-th system as $A_{j}$, I will denote this set of systems as $A=\{A_{j}|j=1,\cdots ,M\}$. The individual systems are not necessarily physically close to each other; they might be in different cities or even on different continents. Systems that are located far apart are unlikely to interact, but there is no reason to exclude them. Note that the set of systems, A, can be equally well viewed as a composite system with internal constraints.

The *j*-th system contains $N_{j}$ particles, which are confined to a volume $V_{j}$ and are governed by a Hamiltonian $H_{j}$. Direct interactions between systems are essential for thermal contact but are assumed to be of negligible magnitude whether or not systems exchange energy. The energy of system *j* will be denoted as $E_{j}$, and the total energy of the entire set A is $E_{T}=\sum_{j=1}^{M}E_{j}$. Similarly, the total number of particles is $N_{T}=\sum_{j=1}^{M}N_{j}$, and the total volume is $V_{T}=\sum_{j=1}^{M}V_{j}$. The total energy, $E_{T}$, volume, $V_{T}$, and particle number, $N_{T}$, are all constants. The generalization to more than one type of particle is straightforward and is discussed in Section 3.6.

The thermodynamic entropy is a function of the equilibrium state of the system. It has certain specific properties, discussed in Section 2.4, that enable the calculation of the new equilibrium values after releasing (or reimposing) any of the constraints on exchanges between systems. The state of a system *j* is specified by the values of $E_{j}$, $V_{j}$, and $N_{j}$. The entropy of system *j*, $S_{j}\left(E_{j},V_{j},N_{j}\right)$, does not depend on any variables other than $E_{j}$, $V_{j}$, and $N_{j}$.

It is essential to thermodynamics that the measurements cannot be made with arbitrary precision, as described in the next subsection.

### 2.2. Limited Experimental Resolution

As Gibbs wrote in the preface to his book on statistical mechanics,

The laws of thermodynamics ...express the laws of mechanics of such systems as they appear to beings who have not the fineness of perception to enable them to appreciate quantities of the order of magnitude of those which relate to single particles, and who cannot repeat their experiments often enough to obtain any but the most probable results.

[6]

I will call a system macroscopic if it has enough particles to make the relative fluctuations too small to observe. This criterion depends on the resolution of the relevant experiments, and to some extent on choices made by the experimenter. This is discussed in Section 3.2. I will only consider finite systems because real systems are finite and it is not necessary to consider the limit of infinite size.

### 2.3. The Purpose of Thermodynamics

The purpose of thermodynamics has been described by Callen as follows.

The single, all-encompassing problem of thermodynamics is the determination of the equilibrium state that eventually results after the removal of internal constraints in a closed, composite system.

[8,9]

The inverse process, in which the equilibrium state is to be determined after the imposition of internal constraints (or the separation of systems) is trivial because the thermodynamic states of the systems do not change.

### 2.4. The Postulates of Thermodynamics

To accomplish its purpose of predicting the thermodynamic behavior after exchanges between systems, the entropy must satisfy certain postulates, which were originally codified by originally codified by Callen [8,9,10,11]. I have simplified these postulates so that they are more generally applicable [13]. The essential postulates are:

Postulate 1: Equilibrium States

There exist equilibrium states of a macroscopic system that are characterized uniquely by a small number of extensive variables.

Postulate 2: Entropy Maximization

The values assumed by the extensive parameters of an isolated composite system in the absence of an internal constraint are those that maximize the entropy over the set of all constrained macroscopic states.

Postulate 3: Additivity

The entropy of a composite system is additive over the constituent subsystems. The entropies of two systems are additive when $S_{A,B}=S_{A}+S_{B}$.

Postulate 4: Continuity and differentiability

The entropy is a continuous and differentiable function of the extensive parameters.

The four essential postulates specify various properties of the entropy. When a function is found that satisfies all four postulates, it is a satisfactory form for the entropy. There are two optional postulates, which are not necessary for a valid entropy function, but which are often satisfied and quite useful [13].

Postulate 5: Extensivity

The entropy is an extensive function of the extensive variables. The entropy of a system is extensive when λS(U,V,N)=S(λU,λV,λN).

This postulate is true only if the system is homogeneous. It forbids adsorbing walls. If it is true, the Euler equation and the Gibbs-Duhem relation are valid [8,9,11].

Postulate 6: Monotonicity

The entropy is a monotonically increasing function of the energy for equilibrium values of the energy.

If this postulate is true, it allows the entropy as a function of energy to be inverted to give the energy as a function of entropy. Legendre transforms then produces the familiar thermodynamic potentials [8,9,11]. Montonicity is, however, not necessary. Massieu functions are less familiar, but no more difficult, produce the usual results for monotonically increasing entropy, and the consistent results for non-monotonic entropy [13,14,15].

Postulate 7: Nernst Postulate

The entropy of any system is non-negative.

The Nernst postulate, also known as the third law of thermodynamics, is only valid for quantum systems. It is not needed in the current discussion.

### 2.5. The Neglect of the Energy Dependence for This Discussion

The original paradoxes were stated in the context of the classical ideal gas, so I will follow this tradition. The examples in this paper will use only classical statistical mechanics in the limit that there are no interactions between particles. For this paper, only the particle-number dependence of the entropy is relevant. Therefore, I will ignore the energy, and restrict the analysis to the configurational degrees of freedom.

### 2.6. The Models Used in This Paper

Since the first paradox involves a comparison between mixing two different gases and mixing two samples of the same gas, I introduce two distinct ideal gases, labeled *a* and *b*. For each system *j*, the number of each type of particle must be specified separately as $N_{a,j}$ and $N_{b,j}$. The total number of each type of particle is $N_{x,T}=\sum_{j=1}^{M}N_{x,j}$, where x=a or x=b.

In the following section, I give a theoretical derivation of the configurational contributions to the entropy based on the theory of probability. In Section 4, I discuss an important detail in the definition of the entropy, before concluding the discussion in Section 5 with the resolution of Gibbs’ paradoxes.

## 3. Definition of Entropy

Definitions of entropy are usually based on the properties of isolated systems in equilibrium. The use of isolated systems is odd because the most important properties of the entropy involve the exchange of energy, volume, or particles between systems. Although Boltzmann began his 1877 paper with explaining the concept of entropy on the basis of the exchange of energy between two systems [4,5], his definition has gone into the general literature as a property of an isolated system. The famous equation on Boltzmann’s tombstone, “S=klogW,” is due to Planck [16], and was put there long after Boltzmann’s death.

Both of Gibbs’ paradoxes involve the exchange of particles between systems, and an understanding of the dependence of the entropy on the particle numbers is crucial. In the first sentence of the abstract of a paper on the Gibbs’ paradox, van Kampen wrote that,

The dependence of the entropy on the number of molecules can never be found from studying closed systems.

[17]

Jaynes made a similar comment a few years later.

As a matter of elementary logic, no theory can determine the dependence of entropy on the size *N* of a system unless it makes some statement about a process where *N* changes.

[18]

I agree with these statements. By considering the exchange of particles between multiple systems, we can determine the particle-number dependence of the entropy.

### 3.1. Exchanging Particles or Volume

The set of systems, A, includes all macroscopic thermodynamic systems that might exchange particles with each other. At the beginning, I assume that each system is perfectly isolated from all the other systems. Later, constraints may be removed or added according to the wishes of the experimenter.

Each system, *j*, has walls that can confine an arbitrary number of particles, $N_{x,j}$, of each type (x=a or x=b) to a volume, $V_{j}$. The total number of each type of particle is given by $N_{x,T}=\sum_{j=1}^{M}N_{x,j}$, and the total volume is $V_{T}=\sum_{j=1}^{M}V_{j}$.

The derivation of the entropy presented below assumes that the pistons exchanging volume between two systems have the same cross section, although that cross section may be different for the pistons linking two other systems. This is not the most general experimental situation. The same form of the entropy will correctly predict equilibrium for the case of pistons with differing cross sections on each side linking the systems [8,9]. This application has nothing to do with Gibbs’ paradox.

### 3.2. Measurable Difference

Particles have traditionally been classified as distinguishable or indistinguishable (or as identical or non-identical), with definitions that have been many and varied [19]. In my opinion, what is necessary to the definition of entropy is whether or not the particles are measurably different.

The example that seems to bring out the essential issue is that of colloidal particles—especially particles containing roughly $10^{9}$ atoms and suspended in a liquid [20,21,22,23,24]. A colloidal suspension can be identified by the Tyndall effect. Such particles are large enough to be well described by classical mechanics, and if they are sufficiently dilute, as a classical ideal gas. It is well known from experimental work that the ideal gas entropy describes such colloids well, but it does need the factor of $1/N_{j}!$. This produces difficulties for traditional explanations that rely on indistinguishability because colloidal particles are not indistinguishable. They have different numbers of atoms, different arrangements of the atoms, and even different types of atoms. They cannot be regarded as indistinguishable or identical, which traditionally would imply that the factor of $1/N_{j}!$ should be missing [20,21,22,23,24].

It is important to note that whether particles are measurably different depends on *the equipment available for the experiments*. An experimenter can also choose not to pay attention to differences that could be measured with a different experimental arrangement. Ignoring these differences would affect the appropriate form of the entropy, but would still yield consistent thermodynamics for that choice of experimental resolution [18].

This approach to defining the thermodynamic entropy might be regarded by some as being subjective, and therefore unsuitable for physics. I disagree with such a point of view. A physical theory should be objective in the sense of giving the same results for different investigators *who have the same information*. This definition provides correct results both before and after the discovery of Whifnium (see Section 3.7 for a discussion of Jaynes’ delightful example), or before and after the discovery of isotopes [18].

### 3.3. Initial Probability Distribution of Particles

Since the behavior of classical particles that are not measurably different from each other is the same for distinguishable and indistinguishable particles, I will use distinguishable particles to calculate the probabilities [25]. Imagine that the $N_{x,T}$ particles of type *x* are numbered, although we are not able to measure the number of any particle to identify it experimentally.

Consider the problem of dividing the $N_{x,T}$ particles (x=a or x=b) among the *M* systems introduced above in Section 3.1. Begin with a single particle, say particle number 1. I assume that the probability of any particle being in system *j* is proportional to the volume $V_{j}$ so that normalization gives the probability equal to $V_{j}/V_{T}$. In the absence of further information, particle 1 could be in any of the *M* systems.

Next consider all $N_{T}$ particles. The total number of particles is given by $N_{T}=N_{a,T}+N_{b,T}$, where $N_{x,T}=\sum_{j=1}^{M}N_{x,j}$. If the particles of a given type have the property that they are not measurably different and they do not interact with other particles, their probability of being in any particular system is independent of the position of any other particle. The probability of a macroscopic state specified by the occupation numbers $\left\{N_{x,j}|j=1,\cdots ,M\right\}$ is then

(1) $$P\left(\{N_{a,j},N_{b,j}\}\right)=\left(\frac{N_{a,T}!}{V_{T}^{N_{a,T}}}\right)\left(\frac{N_{b,T}!}{V_{T}^{N_{b,T}}}\right)\prod_{j=1}^{M}\left[\left(\frac{V_{j}^{N_{a,j}}}{N_{a,j}!}\right)\left(\frac{V_{j}^{N_{b,j}}}{N_{b,j}!}\right)\right]$$

### 3.4. Definition of the Boltzmann Entropy

In the spirit of Boltzmann [4,5], we can define the configurational component of the total entropy of the *M* systems as the logarithm of the probability distribution in Equation (1) [12,13,20,25,26,27,28,29].

(2) $$S_{B}\left(\left\{N_{a,j},N_{b,j}\right\}\right)=k_{B}\ln P\left(\left\{N_{a,j},N_{b,j}\right\}\right)+X,$$

where $k_{B}$ is Boltzmann’s constant (first introduced by Planck [16,30]), and *X* is an arbitrary constant.

When I first wrote about this way of defining the thermodynamic entropy, I only used exchanges between two systems to illustrate the idea [25,26,27,28]. Objections to this approach and its extension to many systems were raised [31,32,33] and answered [12,29]. For completeness, I have included a sketch of the original argument in Appendix A [25].

The value of *X* does not affect any physical prediction of the theory, which is perhaps made more obvious by emphasizing that all possible thermodynamic systems are involved in the derivation, and *M* is an enormous (and unknown) number [12].

Inserting Equation (1) into Equation (2), we find

(3) $$S_{B}\left(\left\{N_{a,j},N_{b,j}\right\}\right)=k_{B}\ln\left[\left(\frac{N_{a,T}!}{V_{T}^{N_{a,T}}}\right)\left(\frac{N_{b,T}!}{V_{T}^{N_{b,T}}}\right)\prod_{j=1}^{M}\left(\frac{V_{j}^{N_{a,j}}}{N_{a,j}!}\right)\left(\frac{V_{j}^{N_{b,j}}}{N_{b,j}!}\right)\right]+X,$$

which can be written as

(4) $$\begin{array}{ccc} S_{B}\left(\left\{N_{a,j},N_{b,j}\right\}\right) & = & k_{B}\left[\ln\left(\frac{N_{a,T}!}{V_{T}^{N_{a,T}}}\right)+\ln\left(\frac{N_{b,T}!}{V_{T}^{N_{b,T}}}\right)\right.\\ & & +\left.\sum_{j=1}^{M}\ln\left(\frac{V_{j}^{N_{a,j}}}{N_{a,j}!}\right)+\sum_{j=1}^{M}\ln\left(\frac{V_{j}^{N_{b,j}}}{N_{b,j}!}\right)\right]+X. \end{array}$$

Collecting the terms that contain only the variables related to an individual system *j* gives

(5) $$S_{B,j}\left(N_{a,j},N_{b,j}\right)=k_{B}\ln\left(\frac{V_{j}^{N_{a,j}}}{N_{a,j}!}\right)+k_{B}\ln\left(\frac{V_{j}^{N_{b,j}}}{N_{b,j}!}\right).$$

This is the configurational part of the Boltzmann entropy of the system *j*. We can write the total Boltzmann entropy of the *M* systems as

(6) $$S_{B}\left(\left\{N_{a,j},N_{b,j}\right\}\right)=\sum_{j=1}^{M}S_{B,j}+k_{B}\ln\left(\frac{N_{a,T}!}{V_{T}^{N_{a,T}}}\right)+k_{B}\ln\left(\frac{N_{b,T}!}{V_{T}^{N_{b,T}}}\right)+X.$$

Since $N_{a,T}$, $N_{b,T}$, $V_{T}$ and *X* are all constants, the last three terms in Equation (6) do not play any role in thermodynamic predictions and may be ignored [12].

The initial conditions for the values of $\left\{N_{a,j},N_{b,j}\right\}$ (and $\left\{E_{j},V_{j}\right\}$ if we consider the full entropy) are arbitrary, subject to the sum rules for $N_{a,T}$ and $N_{b,T}$ (and $E_{T}$ and $V_{T}$), and are determined by the experimenter. To start, all systems are isolated to establish their initial values. Once the initial conditions have been established, the systems can exchange particles or not, as the experimenter decides.

The four essential postulates that the entropy must satisfy are given above in Section 2.4. $S_{B,j}\left(N_{a,j},N_{b,j}\right)$ clearly satisfies the first essential postulate, and the form of Equation (6) confirms that it satisfies the third postulate (additivity). Equation (2) gives the entropy as the logarithm of the probability distribution so that the location of the maximum of the entropy automatically gives the mode of the probability distribution. For a large number of particles, the mode is not experimentally distinguishable from the mean [34]. Therefore, $S_{B,j}\left(N_{a,j},N_{b,j}\right)$ satisfies the first three essential postulates.

The fourth essential postulate presents a difficulty. The number of particles is, by definition, discrete. The usual way to deal with this problem is to ignore it, and this is what I will do in this section. However, in Section 4, I will return to the problem and give a more satisfactory solution.

In the meantime, if Stirling’s approximation $\left(\ln N!\approx N\ln N-N\right)$ is used, the expression for the Boltzmann entropy becomes continuous and easy to work with. Equation (5) becomes

(7) $$S_{B,j}\left(N_{a,j},N_{b,j}\right)=k_{B}N_{a,j}\ln\left(\frac{V_{j}}{N_{a,j}}\right)+k_{B}N_{b,j}\ln\left(\frac{V_{j}}{N_{b,j}}\right)+k_{B}N_{a,j}+k_{B}N_{b,j},$$

and all four essential postulates are satisfied for this approximate form of the entropy.

### 3.5. Exchange of Particles of a Single Kind

Consider the release of the constraint that systems *ℓ* and *m* cannot exchange particles of type *a*, with all other constraints remaining in place. This release would correspond to replacing an impermeable wall between the two systems with a semipermeable membrane that allows particles of type *a* to pass through, but not particles of type *b*. If there are no particles of type *b* in either system, this is the case of only one type of gas.

After allowing particles of type *a* to be exchanged between systems *ℓ* and *m*, the exact value of the location of the mean of the probability distribution for $N_{a,\ell }$ from Equation (1) is known to be

(8) $$\langle N_{a,\ell }\rangle =\left(\frac{N_{a,\ell }+N_{a,m}}{V_{\ell }+V_{m}}\right)V_{\ell }.$$

From Stirling’s approximation to the Boltzmann entropy (Equation (7)), the maximum (or mode) of the probability distribution (or the entropy) can be found easily under the condition that the sum $N_{a,\ell }+N_{a,m}$ is constant. Inserting $N_{a,m}=N_{a,b}-N_{a,\ell }$ into Equation (7) and setting the derivative with respect to $N_{a,\ell }$ equal to zero, gives a very good approximation for the mode of the distribution, which turns out to be the exact mean in Equation (8).

Separating systems *ℓ* and *m* gives no difficulties since $\langle N_{a,m}\rangle$ and $\langle N_{a,\ell }\;\rangle$ are known. However, the probability distribution for the particle numbers now has a width. Since all systems might reasonably be assumed to have exchanged particles with another system sometime in their history, all thermodynamic systems have a width in their probability distributions, and this should be reflected in the entropy. In Section 4, I will introduce (and justify) the grand canonical entropy, which solves that problem. First, I will turn to the problem of mixing two different kinds of particles.

### 3.6. Exchange of Particles of More than One Kind

If the impermeable wall in Section 3.5 is simply removed, particles of both types, *a* and *b*, can be exchanged between systems *ℓ* and *m*. Now, Equation (4) for the relevant part of the entropy (without Stirling’s approximation) becomes

(9) $$S_{B,\mathrm{rel}}=k_{B}\ln\left(\frac{V_{\ell }^{N_{a,\ell }}}{N_{a,\ell }!}\right)+k_{B}\ln\left(\frac{V_{m}^{N_{a,m}}}{N_{a,m}!}\right)+k_{B}\ln\left(\frac{V_{\ell }^{N_{b,\ell }}}{N_{b,\ell }!}\right)+k_{B}\ln\left(\frac{V_{m}^{N_{b,m}}}{N_{b,m}!}\right),$$

and, in addition to Equation (8), there is

(10) $$\langle N_{b,\ell }\rangle =\left(\frac{N_{b,\ell }+N_{b,m}}{V_{\ell }+V_{m}}\right)V_{\ell }.$$

The single equation for one type of gas now becomes two equations.

The cases of one vs. two gases therefore explicitly differ in the form of the entropy. Which form is chosen should be made on the basis of what is measurable.

### 3.7. Other Treatments without Quantum Mechanics

There have been several previous arguments for the factor of 1/N! that did not require the use of quantum mechanics.

Gibbs showed that the entropy of an ideal gas must be extensive to predict the correct behavior [1,6]. In this calculation he used the fact that the ideal gas is an homogeneous system, so his proof really relied on the factor of 1/N!, rather than the extensivity. It has been quite common to restrict consideration to the thermodynamics of homogeneous systems [8,9]. Homogeneity has the advantage of making the Euler equation valid, although it is really not necessary.

Gibbs also presented a derivation of the 1/N! factor in Chapter XV of his book on statistical mechanics [6]. He discussed the difference between using “specific” phases (without the factor of 1/N!) and “generic” phases (with the factor of 1/N!). Gibbs’ derivation was essentially correct, but his reasoning was sufficiently convoluted to lead most people to prefer an explanation based on quantum mechanics.

Gibbs explored the limits of the concept of distinct gases by considering two gases with identical properties except for an attraction to “some other substances” [1]. Jaynes investigated much the same situation by assuming that there might be two types of Argon, which seem identical in all measurable properties. He further assumed that a hypothetical “Whifnium” (“which is so rare that it has not yet been discovered” [18]) would dissolve one type of Argon but not the other. Jaynes predicted that Whifnium would be discovered “in the next Century” [18].

In the example of Jaynes, if an experimenter uses a form of the entropy that corresponds to measurable differences—either with or without Whifnium—the results would be correct. The worst that could be said about treating different gases as if they were the same, is that some phenomena would be missed. However, they would be missed in any case because the difference would not be measurable.

Van Kampen wrote an insightful paper in which he showed how to get the factors of $1/N_{1}!$ and $1/N_{2}!$ by combining two systems [17]. However, van Kampen only kept the factor of $1/N_{1}!$ in the definition of the partition function for system 1 under the assumptions that system 2 became infinitely large. He also assumed that the two systems remained open to the exchange of particles. van Kampen’s demonstration contained an error in the assumption that both the pressure and the temperature were fixed while the system was connected to an infinite particle reservoir. Under such conditions, the number of particles in the system is undetermined.

In 1992, Jaynes demonstrated that the definition of entropy depended on the state of knowledge of the experimenter [18]. He considered the effects of two imaginary elements he called Whifnium and Whoofnium. Jaynes showed that experiments that did not use these elements and did not include their effects in the entropy functions used to analyze these experiments were still capable of giving correct and consistent results.

Warren maintains the traditional expression for the partition function without the factor of 1/N!. However, he notes that when two systems are considered together, “one must sum over the $N!/N_{1}!N_{2}!$ ways of partitioning the particles” [22]. Unfortunately, he stops short of using that fact as a basis for developing a new definition of entropy. His basic reason for including the factor of 1/N! is to obtain an extensive expression for the entropy.

Frenkel argues that the explanation of Gibbs’ paradox does not depend on quantum mechanics [21], citing both Warren [22] and myself [20,27]. Our justifications of the factor of 1/N! are, of course, different, but our results are consistent with each other.

Sethna introduced the concept of “undistinguished” particles, by which he apparently meant the same thing I mean by not measurably different [35]. He advocated dividing the partition function for *N* undistinguished particles by N!.

Cates and Manoharan commented that,

First, not all reasonable-sounding definitions of entropy for classically distinguishable particles are equivalent: some are right and some are wrong. Second, experiments on colloidal suspensions can resolve with striking clarity what the right definitions are.

[23]

They discuss several suggested approaches to entropy based on subjective and objective definitions [23]. They come to the conclusion that, “the informatic view is the simplest way to interpret experiments on colloids,” but do not advocate any particular definition as being generally correct.

## 4. Refining the Definition of Entropy

Up to this point, I have been ignoring the energy dependence of the entropy. Although it is not directly relevant to the main question, its inclusion is natural for the next step. If we release a constraint and allow two systems to exchange particles, they will also inevitably exchange energy. Just as the width of the particle-number distribution must be non-zero after an exchange of particles, so must the width of the energy distribution be non-zero.

In both cases, the widths can be calculated in the grand canonical ensemble, which is clearly correct if the system has interacted with one that is much bigger. As I will explain in the next subsection, even when the system has interacted with a smaller system, the grand canonical entropy is valid [12].

### 4.1. Justification of the Grand Canonical Entropy

If system *X* has exchanged particles with another system *Y* at any time in its history, then its particle-number probability distribution (as well as its energy distribution) has a non-zero width. If system *Y* was much larger than system *X*, the particle-number probability distribution of *X* is given by the grand canonical ensemble. The entropy of *X* can be evaluated in the grand canonical ensemble.

If *Y* was of comparable size or smaller than *X*, the width of the particle-number probability distribution of *X* is smaller than that given by the grand canonical ensemble [36]. However, the entropy is still equal to its grand canonical value [12].

To see this, consider three systems, *A*, *B*, and *C*, which differ in volume. Systems *A* and *B* are equal in volume, $V_{A}=V_{B}$, but $V_{C}>>V_{A}+V_{B}$. Let all three systems exchange particles, so that the number densities of the three systems are the same.

(11) $$\frac{\langle N_{A}\rangle }{V_{A}}=\frac{\langle N_{B}\rangle }{V_{B}}=\frac{\langle N_{C}\rangle }{V_{C}}$$

The particle-number probability distributions of systems *A* and *B* are given by the grand canonical ensemble, and the entropies of *A* and *B* are also calculated in the grand canonical ensemble.

Now close system *C* and separate it from *A* and *B*. The entropies of the systems must remain unchanged. If they decreased, it would violate the second law. If they increased, returning to the original position would lower the entropy, which would also violate the second law. Therefore, the entropies must remain the same.

However, with *C* separated from *A* and *B*, the two smaller systems are only in equilibrium with each other. Their particle-number probability distributions are narrower than when they were exchanging particles with *C*, but their entropies are unchanged.

No thermodynamic measurement can determine the size of the last system with which a given system has exchanged particles. For all macroscopic systems, the entropy is given by the grand canonical expression.

### 4.2. The Grand Canonical Entropy of the Classical Ideal Gas

The grand canonical entropy of the classical ideal gas has been calculated exactly in a previous paper [13]. It is found to be exactly extensive, without using Stirling’s approximation. This may be surprising to some, but it is quite reasonable since ideal gas particles are completely independent of each other. An outline of the calculation is given in Appendix B.

It is trivial to extend the expression to the case of two different kinds of particles. The entropy of the system *j* is a function of the average particle numbers $\langle N_{a,j}\rangle$ and $\langle N_{b,j}\rangle$, and the average energy $U_{j}=\langle E_{j}\rangle$.

(12) $$\begin{array}{ccc} S_{GC,j} & = & \langle N_{a,j}\rangle k_{B}\left[\frac{3}{2}\ln\left(\frac{U_{j}}{\langle N_{a,j}\rangle }\right)+\ln\left(\frac{V_{j}}{\langle N_{a,j}\rangle }\right)+\ln{\left(\frac{4\pi m}{3h^{2}}\right)}^{3/2}+\frac{5}{2}\right]\\ & & +\;\langle N_{b,j}\rangle k_{B}\left[\frac{3}{2}\ln\left(\frac{U_{j}}{\langle N_{b,j}\rangle }\right)+\ln\left(\frac{V_{j}}{\langle N_{b,j}\rangle }\right)+\ln{\left(\frac{4\pi m}{3h^{2}}\right)}^{3/2}+\frac{5}{2}\right] \end{array}$$

## 5. Resolution of the Paradoxes

The resolution of Gibbs’ paradoxes is now straightforward. The main feature of the resolution is that the entropy should be defined in terms of the probability of transferring particles (or energy, or volume) between systems. This was first argued by Boltzmann, who even included the word “Wahrscheinlichkeitsrechnung” (probability calculation) in his title [4,5]. Planck missed an important point by trying to apply Boltzmann’s ideas to an isolated system [30]. I believe that the definition of the classical entropy as I have presented it in this paper and elsewhere represents Boltzmann’s intentions.

### 5.1. The First Gibbs’ Paradox

The first Gibbs’ paradox concerns the question of why mixing two volumes of the same gas does not produce an increase in the entropy while mixing two different gases does.

It is useful to consider the probability distribution of the particles when mixing of two volumes of the same gas. To be specific, assume that the two systems, *ℓ* and *m*, have no type *b* particles and the same energy density and number density of type *a* particles, that is

(13) $$\frac{U_{a,\ell }}{\langle N_{a,\ell }\rangle }=\frac{U_{a,m}}{\langle N_{a,m}\rangle },$$

and

(14) $$\frac{\langle N_{a,\ell }\rangle }{V_{\ell }}=\frac{\langle N_{a,m}\rangle }{V_{m}}.$$

Remove the constraint between the two systems, *ℓ* and *m*, so that the two volumes of gas can mix. After they have come to equilibrium, separate the two systems.

As described in Section 2, we originally assumed that any particle could be anywhere in the *M* systems, which were scattered throughout the world. The probability of being found in system *ℓ* was given by $V_{\ell }/V$. This assumption seems to be reasonable, since we have assumed that no measurement can determine which system it is in. After the “mixing” of two gases, *the probability distribution is unchanged*. Mixing two samples of the same gas has no effect at all; the probability of any particular particle being in system *ℓ* is exactly the same before and after mixing. It is only to be expected that the total entropy is unchanged.

The mixing of different kinds of gases has an effect on the probability distribution. We have assumed that they are measurably different, so that we can set up an experiment with only particles of type *a* in system *ℓ* and only particles of type *b* in system *m*. For simplicity, assume that $V_{\ell }=V_{m}=V_{o}$ where $V_{o}$ is a constant. Similarly, assume that $\langle N_{a,\ell }\rangle /V_{\ell }=\langle N_{b,m}\rangle /V_{m}=N_{o}/V_{o}$, with $\langle N_{b,\ell }\rangle =\langle N_{a,m}\rangle =0$. For simplicity, ignore the energy dependence in Equation (12) and the constant $\ln{\left(4\pi m/3h^{2}\right)}^{3/2}+5/2$.

Before mixing, the relevant terms in the entropy of the two systems (ignoring the other M−2 systems) are

(15) $$S_{GC,\mathrm{before}}=N_{o}k_{B}\ln\left(\frac{V_{o}}{N_{o}}\right)+N_{o}k_{B}\ln\left(\frac{V_{o}}{N_{o}}\right)=2N_{o}k_{B}\ln\left(\frac{V_{o}}{N_{o}}\right)$$

where the first term comes from the entropy of system *ℓ* and the second from the entropy of system *m* (both have the same magnitude).

After mixing, a simple calculation gives the new entropy as

(16) $$S_{GC,\mathrm{after}}=\frac{N_{o}}{2}k_{B}\ln\left(\frac{V_{o}}{N_{o}/2}\right)+\frac{N_{o}}{2}k_{B}\ln\left(\frac{V_{o}}{N_{o}/2}\right)+\frac{N_{o}}{2}k_{B}\ln\left(\frac{V_{o}}{N_{o}/2}\right)+\frac{N_{o}}{2}k_{B}\ln\left(\frac{V_{o}}{N_{o}/2}\right)$$

or

(17) $$S_{GC,\mathrm{after}}=2N_{o}k_{B}\ln\left(\frac{V_{o}}{N_{o}/2}\right)=2N_{o}k_{B}\left[\ln\left(\frac{V_{o}}{N_{o}}\right)+\ln2\right]$$

The increase in entropy is *exactly* as expected. The experimentally observed state has changed.

### 5.2. The Second Gibbs’ Paradox

The second Gibbs’ paradox is due to the apparent difficulty in explaining the factor of $1/N_{j}!$ in the expression for the entropy of system *j*. The paradox is based on an expression for the entropy that Boltzmann derived for the energy dependence alone. He had explicitly stated that the number of particles was assumed constant [4,5]. That form of the entropy was not suitable for determining the particle-number dependence; Boltzmann never claimed that it was.

The second Gibbs’ paradox is not a paradox at all if the entropy is derived from the properties of a set of systems that can exchange particles, as it is in this paper. It follows immediately from the multinomial distribution in Equation (1), which has a factor of $N_{j}!$ for every system in the denominator.

The idea of exchanging energy between two systems and associating the entropy with the maximum of the probability distribution was given in Boltzmann’s 1877 paper [4,5]. Extending it to the exchange of particles is straightforward.

I have given a derivation of the entropy $S_{j}$ for system *j* that Boltzmann could have given in the 19th century. The derivation includes the correct factors of $1/N_{x,j}!$. No quantum mechanics was used in the derivation.

## 6. Conclusions

The resolution of Gibbs’ first paradox is subtle. Gibbs understood the impossibility of returning a mixture of like gases to the original containers. However, he assumed that it was meaningful to speak of the original containers for each gas. Gibbs assumed that particles somehow took on the identity of the system that they had been in at the start of an experiment. Actually, if the particles cannot be identified at the end of an experiment because they are not measurably different, they certainly could not have been identified before any measurements had been made. The probability distribution of the particles is given by Equation (1) both before and after mixing. Mixing particles that are not measurably different has no effect at all.

The Gibbs’ second paradox is easier to understand because it is a consequence of the probability distribution given in Equation (1). The message to be drawn from these calculations is that Boltzmann would have saved physicists a great deal of trouble if he had applied his interpretation of the entropy to calculate the particle-number dependence. It is far simpler than his calculation of the energy (or temperature) dependence and would have dispensed with Gibbs’ second paradox during the 19th century. There was no need to wait for the invention of quantum mechanics, which plays no role in the explanation of either of Gibbs’ paradoxes.

## Appendix A. Derivation of the Entropy Using Two Systems

If we only use two systems, we can still derive the entropy, including the factor of $1/N_{j}!$. In fact, this is the way I first presented the derivation [20,25]. I will limit the argument to one type of particle for simplicity. I will also ignore the energy dependence of the entropy, as I have in the rest of this paper.

Consider two systems that could potentially exchange particles, except for constraints that might prevent such exchanges. These constraints can be released or reimposed as the experimenter decides. There are initially $N_{1}$ particles in system 1 and $N_{2}$ particles in system 2. $N_{1}$ and $N_{2}$ are variables because the two systems can potentially exchange particles. The total number of particles, $N=N_{1}+N_{2}$, is constant because the two systems are isolated from the rest of the universe. System 1 has a volume $V_{1}$ and system 2 has a volume $V_{2}$.

If particle exchange is forbidden, the initial values of $N_{1}$ and $N_{2}$ can be anything. If the two systems are allowed to exchange particles and each particle has a uniform probability density of being anywhere in the total volume $V=V_{1}+V_{2}$, the probability of finding $N_{1}$ particles in system 1 and $N_{2}$ particles in system 2, would be given by

(A1) $$P\left(N_{1},V_{1};N_{2},V_{2}\right)=\frac{N!}{N_{1}!N_{2}!}\frac{V_{1}^{N_{1}}V_{2}^{N_{2}}}{V^{N}}.$$

To determine the equilibrium values, $N_{1}^{*}$ and $N_{2}^{*}$, maximize $P(N_{1},V_{1};N_{2},V_{2})$, subject to the condition that *N* is constant. If the constraint forbidding particle exchange is subsequently reimposed, the values $N_{1}=N_{1}^{*}$ and $N_{2}=N_{2}^{*}$ are retained.

I defined the configurational component of the total entropy of two systems as the logarithm of the particle probability distribution [12,13,20,25,26,27,28,29], plus an arbitrary additive constant. The total configurational entropy of the two systems then

(A2) $$S\left(N_{1},V_{1};N_{2},V_{2}\right)=k_{B}\ln P\left(N_{1},V_{1};N_{2},V_{2}\right)+X,$$

where *X* is an arbitrary constant, and the maximum of the entropy gives the equilibrium values of $N_{1}$ and $N_{2}$. The entropy can be written

(A3) $$S\left(N_{1},V_{1};N_{2},V_{2}\right)=k_{B}\ln\left(\frac{V_{1}^{N_{1}}}{N_{1}!}\right)+k_{B}\ln\left(\frac{V_{2}^{N_{2}}}{N_{2}!}\right)-k_{B}\ln\left(\frac{V^{N}}{N!}\right)+X$$

The last two terms are, of course, constant. Their values have no physical consequences. At this point, I chose $X=k_{B}\ln\left(V^{N}/N!\right)$ to simplify the equation.

(A4) $$S\left(N_{1},V_{1};N_{2},V_{2}\right)=k_{B}\ln\left(\frac{V_{1}^{N_{1}}}{N_{1}!}\right)+k_{B}\ln\left(\frac{V_{2}^{N_{2}}}{N_{2}!}\right)=S_{1}\left(N_{1},V_{1}\right)+S_{2}\left(N_{2},V_{2}\right)$$

The maximum of this expression still determines the equilibrium values of $N_{1}$ and $N_{2}$ when the particle-number constraint is released under the condition that *N* and *V* are constant.

Since the entropy function for system 1, $S_{1}\left(N_{1},V_{1}\right)$, does not depend in any way on the properties of system 2, system 2 could be replaced by any other system along with its entropy function. Indeed, any two systems could replace systems 1 and 2. Equilibrium conditions after the release of any constraint and subsequent equilibration of the systems (holding the sum of the particle numbers from the new pair of systems constant) would be found from the entropy functions without further calculations in statistical mechanics. In this way, the expressions for the systems’ entropies would satisfy the thermodynamic postulates for any set of systems.

## Appendix B. The Grand Canonical Entropy of the Ideal Gas

This appendix gives the mathematics behind the perfectly extensive grand canonical entropy of the classical ideal gas. The derivation requires the complete expression for the entropy, including the energy dependence. Throughout this derivation, the subscript *j*, labelling the system, will be dropped. It is useful to first calculate the canonical entropy.

### Appendix B.1. The Canonical Entropy

To find the canonical entropy, we first calculate the canonical partition function in the usual way.

(A5) $$Z=\frac{1}{h^{3N}N!}V^{N}\int_{-\infty }^{\infty }d^{3N}p\;\exp\left[-\beta \sum_{j=1}^{3N}p_{i}^{2}/2m\right]=\frac{1}{N!}V^{N}{\left(\frac{2\pi m}{\beta h^{2}}\right)}^{3N/2}$$

For convenience, define a dimensionless entropy as $\tilde{S}=S/k_{B}$ so that the the differential form of the fundamental equation is

(A6) $$d\tilde{S}=\beta \;dU+\left(\beta P\right)dV-\left(\beta \mu \right)dN,$$

where *P* is the pressure, and μ is the chemical potential.The parentheses around (βP) and (βμ) are reminders that these quantities are each to be treated as single variables.

Massieu functions give us a convenient way to derive the entropy. Defining the Legendre transform of the entropy with respect to β as the first Massieu function, we have from Equation (A6),

(A7) $$\beta ={\left(\frac{\partial \tilde{S}}{\partial U}\right)}_{V,N}.$$

The Legendre transform (Massieu function) of $\tilde{S}$ with respect to β is given by

(A8) $$\tilde{S}\left[\beta \right]=\tilde{S}-\beta U=-\beta \left(U-TS\right)=-\beta F,$$

so that

(A9) $$\tilde{S}\left[\beta \right]=\ln Z\left(\beta ,V,N\right).$$

inserting the expression for *Z* from Equation (A5) gives

(A10) $$\tilde{S}\left[\beta \right]=\ln\left[\frac{1}{N!}V^{N}{\left(\frac{2\pi m}{\beta h^{2}}\right)}^{3N/2}\right].$$

Using

(A11) $$-U={\left(\frac{\partial \tilde{S}\left[\beta \right]}{\partial \beta }\right)}_{V,N}$$

we can easily derive

(A12) $$\beta =\frac{3N}{2U}$$

This equation is exact, while the usual Boltzmann entropy gives a tiny error of order 1/N.

To obtain $\tilde{S}$ from $\tilde{S}\left[\beta \right]$, simply invert Equation (A8) Writing this out gives the canonical entropy.

(A13) $$S_{C}=k_{B}\left[N\left(\frac{3}{2}\right)\ln\left(\frac{U}{N}\right)+\ln\left(\frac{V^{N}}{N!}\right)+N\left(\frac{3}{2}\right)\ln\left(\frac{4\pi m}{3h^{2}}\right)+\frac{3N}{2}\right]$$

Only the term involving the volume *V* contains a factor of 1/N!. Note that this expression uses the average energy *U* instead of *E*.

### Appendix B.2. The Grand Canonical Entropy

For the grand canonical ensemble, a second Massieu function S[β,(βμ)] is needed, which uses the equation

(A14) $$-\left(\beta \mu \right)={\left(\frac{\partial \tilde{S}}{\partial N}\right)}_{U,N},$$

in addition to Equation (A7).

The Legendre transform of $\tilde{S}$ with respect to both β and (βμ) is given by

(A15) $$\tilde{S}\left[\beta ,\left(\beta \mu \right)\right]=\tilde{S}-\beta U+\left(\beta \mu \right)N=\ln Z\left(\beta ,V,\left(\beta \mu \right)\right),$$

where Z is the grand canonical partition function.

To obtain the grand canonical partition function, multiply the canonical partition function by exp[(βμ)N] and sum over *N*.

(A16) $$Z=\sum_{N=0}^{\infty }\frac{1}{N!}{\left(V{\left(\frac{2\pi m}{\beta h^{2}}\right)}^{3/2}\right)}^{N}\exp\left[\left(\beta \mu \right)N\right]$$

The series sums to an exponential, eliminating the factor of N!.

(A17) $$Z=\exp\left[\left(V{\left(\frac{2\pi m}{\beta h^{2}}\right)}^{3/2}\right)\exp\left[\left(\beta \mu \right)\right]\right]$$

Using Equation (A15) gives

(A18) $$\tilde{S}\left[\beta ,\left(\beta \mu \right)\right]=\left(V{\left(\frac{2\pi m}{\beta h^{2}}\right)}^{3/2}\right)\exp\left[\left(\beta \mu \right)\right]$$

The usual equation for *N* gives

(A19) $$N={\left(\frac{\partial \tilde{S}\left[\beta ,\left(\beta \mu \right)\right]}{\partial (\beta \mu )}\right)}_{\beta ,V}=\left(V{\left(\frac{2\pi m}{\beta h^{2}}\right)}^{3/2}\right)\exp\left[\left(\beta \mu \right)\right]$$

We can use this equation to solve for (βμ).

(A20) $$\left(\beta \mu \right)=\ln\left[\frac{N}{V}{\left(\frac{\beta h^{2}}{2\pi m}\right)}^{3/2}\right]$$

Taking derivative of $\tilde{S}\left[\beta ,\left(\beta \mu \right)\right]$ with respect to β,

(A21) $$-U={\left(\frac{\partial \tilde{S}\left[\beta ,\left(\beta \mu \right)\right]}{\partial (\beta )}\right)}_{V,(\beta \mu )},$$

gives

(A22) $$U=\frac{3}{2}\beta^{-1}N.$$

Finally, the grand canonical entropy is

(A23) $$S_{GC}=\langle N\rangle k_{B}\left[\frac{3}{2}\ln\left(\frac{U}{\langle N\rangle }\right)+\ln\left(\frac{V}{\langle N\rangle }\right)+\ln{\left(\frac{4\pi m}{3h^{2}}\right)}^{3/2}+\frac{5}{2}\right].$$

Note that this expression is a function of 〈N〉 instead of *N*. This expression for the entropy of a classical ideal gas is exactly extensive and no use has been made of Stirling’s approximation.

References
